# In-Plane Strain Measurement in Composite Structures with Fiber Bragg Grating Written in Side-Hole Elliptical Core Optical Fiber

**DOI:** 10.3390/ma15010077

**Published:** 2021-12-23

**Authors:** Karol Wachtarczyk, Paweł Gąsior, Jerzy Kaleta, Alicja Anuszkiewicz, Marcel Bender, Ralf Schledjewski, Paweł Mergo, Tomasz Osuch

**Affiliations:** 1 Department of Mechanical Engineering, Wrocław University of Science and Technology, Smoluchowskiego 25, 50-370 Wroclaw, Poland; pawel.gasior@pwr.edu.pl (P.G.); jerzy.kaleta@pwr.edu.pl (J.K.); 2 Faculty of Electronics and Information Technology, Institute of Electronic Systems, Warsaw University of Technology, Nowowiejska 15/19, 00-665 Warsaw, Poland; alicja.anuszkiewicz@pw.edu.pl (A.A.); tomasz.osuch@pw.edu.pl (T.O.); 3 Institute of Microelectronics and Photonics, Lukasiewicz Research Network, al. Lotników 32/46, 02-668 Warsaw, Poland; 4 Processing of Composites Group, Montanuniversität Leoben, Otto Glöckel-Straße 2/III, 8700 Leoben, Austria; marcel.bender@unileoben.ac.at (M.B.); ralf.schledjewski@unileoben.ac.at (R.S.); 5 Faculty of Chemistry, Institute of Chemical Sciences, Maria Curie-Skłodowska University, pl. M. Curie-Skłodowskiej 3, 20-031 Lublin, Poland; pawel.mergo@mail.umcs.pl; 6 National Institute of Telecommunications, Szachowa 1, 04-894 Warsaw, Poland

**Keywords:** fiber optic sensor, fiber Bragg grating, high birefringent fibers, side-hole optical fiber, carbon fiber reinforced polymer, multi-axial strain sensing

## Abstract

In this paper, the application of a fiber Bragg grating written in a highly birefringent side-hole elliptical core optical fiber for two-axial strain measurement is presented. Hybrid optical fiber structures achieved by combining large side-holes and elliptical core result in a very high birefringence of 1 × 10^−^^3^ and thus high initial Bragg peak spectral separation of 1.16 nm, as well as a very high transverse force sensitivity, of up to 650 pm/(N/mm) or even −1150 pm/(N/mm), depending on the fiber orientation with respect to the applied force. Due to the ~22 %m/m GeO_2_ concentration in the core the fiber being highly photosensitive, which significantly simplifies FBG fabrication by UV illumination without the need for prior hydrogen loading, which worsens thermal stability. Finally, the developed FBGs written in the highly birefringent side-hole elliptical core optical fiber were embedded in the square composite plates and applied for strain measurements. Tests of two-directional four-point bending have shown usability of such FBG for two-axial in-plane strain measurement with a single FBG in iso-thermal conditions.

## 1. Introduction

Fiber-reinforced polymers (FRPs) have received increasing attention over the last few decades. Due to their good mechanical performance, their usage in transportation and civil engineering is widely known. In modern aircrafts, such as the Boeing 787, half of the weight of the plane is composite [1]. For the safe operation of composite structures, Structural Health Monitoring (SHM) systems are applied. SHM allows for longer safe operation and prediction of composite structure failure due to continuous measurements that provide information on the state of the structure [1,2,3].

In recent years, much effort has been devoted to the application of fiber optic sensors (FOS) to structural health monitoring (SHM), especially composite structures. Fiber optic sensors exhibit many advantages over their electronic counterparts, such as ease of multiplexing, immunity to the electromagnetic interference, and ability to operate in a chemically aggressive, explosive, humid, or dusty environment. Moreover, due to their compact size and shape, they can be successfully integrated into fiber-reinforced polymers (FRP) without compromising their mechanical strength [2,3]. One of the most popular fiber optic sensors are those based on fiber Bragg gratings (FBG), which are most often used for the measurement of basic mechanical quantities, such as strain [4], transverse load [5], and acceleration [6]. In most FBG-based sensors the measured value is derived from Bragg wavelength shift, which makes measurement very accurate and optical power fluctuations insensitive.

Several examples of usage of the FBG sensors written in standard single mode fibers (SMF) and their integration with composite materials for SHM applications can be found in the literature [1,7,8,9,10,11]. FBG sensors were integrated for monitoring of high pressure hydrogen composite vessels [12,13], blades of wind turbine [9], fast patrol boat [14], or a component of an aircraft [10].

The Bragg gratings written in standard single mode fibers are widely used for axial strain measurement. However, the possibility of transverse load measurement has also been analyzed [15]. In this case, birefringence of SMF is induced when transverse load is applied. With the increase in applied force, the Bragg wavelength peak in spectral response begins to split into two separate maxima corresponding to the orthogonal polarizations. The main drawbacks of SMF-based FBG transverse load sensors are low sensitivity, with no directional response, and the difficulties with small values measurement. Below a certain level of the applied force, the spectral response of FBG has one peak, and it is not possible to determine the difference between the Bragg wavelengths corresponding to both orthogonal polarizations [15]. On the other hand, birefringence induced in SMF during the composite curing was used for the simultaneous measurement of multiaxial strain [16] and strain-temperature [17].

Ensuring sufficiently large Bragg peak separation can be easily obtained by FBG inscription in high birefringent (HiBi) optical fibers. In this case, due to the relatively high fiber birefringence, Bragg wavelength peaks corresponding to fast and slow optical axes are well separated, even if no force is applied. Furthermore, due to the lack of axial symmetry in HiBi fibers, the sensitivity to transverse load depends on the orientation of the fiber with respect to the direction of the applied force [18,19]. Various solid HiBi fiber structures, such as Panda, bow-tie, elliptical core, and elliptical cladding, with in-written gratings, have been analyzed as transverse load sensors. In all mentioned fibers, the transverse load sensitivity was quite moderate and does not exceed 230 pm/(N/mm) [20]. Further improvement of Bragg wavelength separation was possible due to the specially designed microstructured optical fibers (MOFs) for grating inscription. Geernaert et al. used a fiber with a microstructure that consisted of three rows of air holes and a central Germanium-doped core. Birefringence of B = 8 × 10^−4^ at λ = 1550 nm results in a Bragg wavelength separation of 0.87 nm when the unpolarized light was used. However, the measured transverse load sensitivity was as low as 90 pm/(N/mm) [21]. Such low sensitivity is caused by the optical fiber construction. Although the air holes of the microstructure can induce stress concentrations in the core region that result in a specific distribution of the material birefringence and makes the fiber polarization maintaining, this air holes topology seems to be suboptimal for producing high stress concentration in the area of fiber center when the transverse load is applied. Transverse load sensitivity was significantly improved by up to 390 pm/(N/mm) when an MOF that had a “butterfly”-like topology of air holes was used for FBG fabrication. Moreover, the initial Bragg wavelength separation (without applied force) was as high as 2.17 nm [22]. Sensitivity enhancement of FBG-based sensors to transverse stress was also tested for side-hole optical fibers [23,24]. In both experiments, higher sensitivities of 212 pm/(N/mm) and 699 pm/(N/mm) were recorded when the two air holes were oriented perpendicular to the applied load. On the other hand, low birefringence of analyzed side-hole fibers causes a dead zone in the transverse load measurement range due to spectral overlapping of polarization-dependent Bragg wavelength peaks.

In this paper, we applied a highly photosensitive side-hole elliptical core optical fiber that has an in-written fiber Bragg grating for an efficient transverse load sensor and for strain sensing. The advantage and novelty of the proposed solution is the hybrid optical fiber structures achieved by combining large side-holes and an elliptical core. Due to such a design, the very high birefringence of 10.5 × 10^−4^ was obtained, which results in a well-separated spectrum of in-written fiber Bragg gratings, even when no force is applied. Furthermore, the fiber is highly photosensitive, as it contains ~22 %m/m GeO_2_ in the core, which allows for effective FBG inscription using UV lasers without prior hydrogen loading and without the need for precise alignment of air holes with respect to the writing beam. This, in turn, considerably simplifies grating fabrication and improves thermal stability. Finally, the developed FBGs written in highly birefringent side-hole elliptical core optical fiber were embedded in composite material and applied for strain measurements.

The paper is structured as follows. Section 2 describes used side-hole elliptic core optical fiber and FBG fabrication, as well as presents the results of both fiber and grating characterization. In Section 3, the transverse force sensing properties of FBG are examined. The obtained results in comparison with previous sensing solutions are discussed. The next section is devoted to the application of fabricated FBG sensors for the measurement of the strain of composites. Finally, the last section summarizes and concludes this work.

## 2. Fiber Bragg Grating Inscribed in Highly Birefringent Side-Hole Fibers

### 2.1. Birefringent Fibers Used for Bragg Grating Inscription

**Figure 1:**
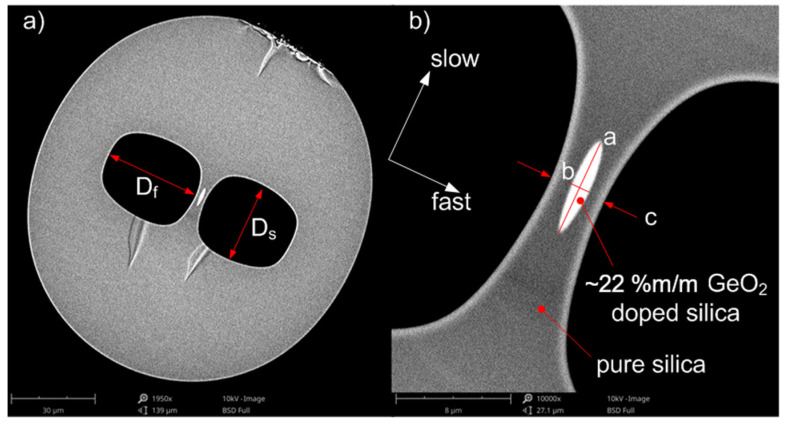
SEM images of SH2 cross-section: (**a**) entire optical fiber, (**b**) zoomed glass bridge with highly GeO_2_-doped silica elliptical core. The coordinate system (white arrows) indicates the slow and fast axes of the SH fiber. The letters *a, b* and *c* indicate long *a* and short *b* diameters of the core ellipse and glass bridge thickness *c*, respectively.

In-house highly birefringent (HiBi) fibers [25] that had two large side-holes and an elliptical core located in a thin glass bridge between the holes were used for FBG inscription, Figure 1. The SH fiber’s preform was stacked of pure silica rods and tubes and cylindrical core preform and had a central area highly doped with germanium (~22 %m/m) and a thin pure silica pad. The fiber preform was then thermally integrated and drawn in a temperature of around 2175 °C and a drawing speed of around 20 m/min. To form the elliptical core of the SH fibers, the elevated pressure was applied to the side-holes, allowing to squeeze the glass bridge along a fast axis. By differentiating pressure in the holes, two fibers were fabricated of the same preform. These types of fibers are labeled side-hole fiber 1 (SH1) and side-hole fiber 2 (SH2). We indicated, using a white coordinate system, the slow (s) and fast (f) axes of the fiber. Along the main coordinates, the fiber cladding diameters (2R), side-hole sizes (D), and elliptical core diameters (a and b) as well as glass bridge thickness (c) were measured in the fast axis direction. The ellipticity of the core (e), which shows the main difference between the SH fibers, was also measured. Higher ellipticity of the core guarantees higher modal birefringence of the fiber with the same material composition [26], therefore we expect it for SH2. The geometric parameters measured directly from the SEM images of the SH fibers are gathered in Table 1. The geometric parameters of the fiber were preserved with an uncertainty below 1%. The glass bridge is thicker for SH1, because the side-holes were not enlarged more during fiber draw. This is also the reason for the smaller core ellipticity and expected birefringence for SH1. Both fibers are of a similar diameter; however, both are elliptical in shape.

### 2.2. Birefringence Measurement

For both fibers, phase (B) and group (G) birefringence was measured using the standard wavelength scanning method [27], combined with the lateral force method [28]. Phase birefringence B is defined as a difference in effective refractive indices of the slow and fast polarization mode of the HiBi fiber [29]:

(1) $$B=n_{\mathrm{eff}}^{s}-n_{\mathrm{eff}}^{f}$$

whereas group birefringence (G) is described by the following formula:

(2) $$G=B-\lambda \frac{\partial B}{\partial \lambda }$$

**Figure 2:**
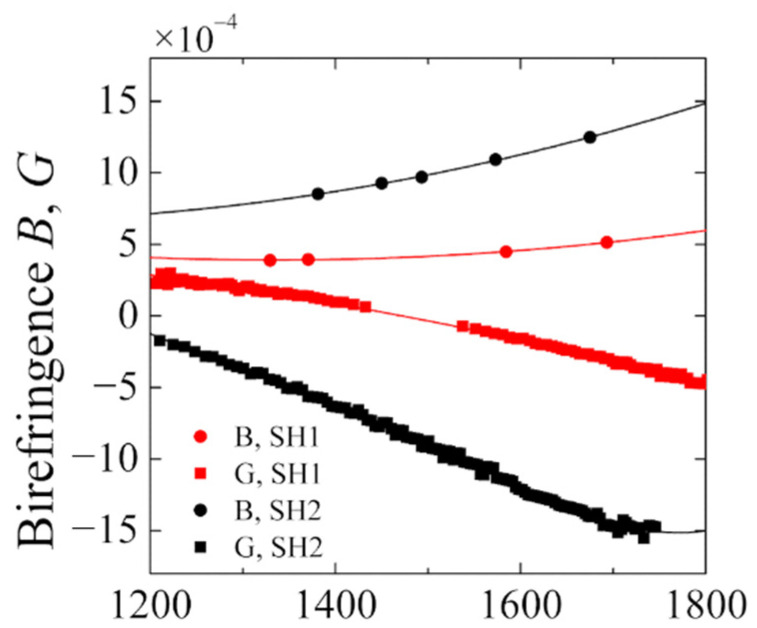
Measured phase B and group G birefringence of the side-hole fibers.

For both fibers, B and G characteristics in near infrared spectral range were measured (Figure 2). Due to the higher core ellipticity and thinner glass bridge where the core is placed, the SH2 fiber has higher birefringence than SH1. The phase birefringence value measured at 1550 nm equals to 10.5 × 10^−4^ and 4.4 × 10^−4^ for SH2 and SH1, respectively.

### 2.3. Bragg Gratings Inscription

Due to the high ~22 %m/m GeO_2_ concentration in the cores, both SH1 and SH2 optical fibers are photosensitive. This means that an additional photosensitivity enhancement, such as hydrogen loading, is unnecessary. Prior to the gratings inscription, short sections of standard SMF-28 optical fibers were spliced to the SH1 and SH2 ends, to ensure compatibility with measurement instruments. The mismatch between the sizes and shapes of the mode fields of spliced fibers resulted in coupling loss that did not exceed 3 dB. Although this value is relatively high as compared with SMF to SMF splicing loss, it does not have a significant impact on the measurement capabilities during both characterization and operation of the HiBi FBG-based sensor.

Fiber Bragg gratings were written using a phase mask technique that ensures the best repeatability among all FBG writing methods [26]. Short 20 ns pulses were emitted by a KrF excimer laser that operates at 248 nm wavelength with a repetition rate 200 Hz. The UV beam was focused on optical fiber through a cylindrical lens with a focal length of 17.5 cm, which produces fiber Bragg gratings with a length of a few mm. Phase mask optimized for illumination with the wavelength interest with a period of 1069 nm was used to ensure Bragg wavelength peaks within the spectral range c.a. 1550 nm, according to the Bragg condition [30]:

(3) $$\lambda_{\mathrm{FBG}}=2n_{\mathrm{eff}}\Lambda =n_{\mathrm{eff}}\Lambda_{\mathrm{PM}}$$

where n_eff_ is the effective refractive index of the mode and Λ stands for the FBG period, and Λ_PM_ is the phase mask period. It is worth mentioning that Equation (3) must be considered separately for slow and fast components of the mode of birefringent fiber, as the Bragg wavelengths will be different for each polarization:

(4) $$\lambda_{\mathrm{FBG}}^{s}=2n_{\mathrm{eff}}^{s}\Lambda \;\mathrm{and}\;\lambda_{\mathrm{FBG}}^{f}=2n_{\mathrm{eff}}^{f}\Lambda .$$

Using Equations (4) and (1), the separation between the peaks can be calculated from the formula:

(5) $$\Delta \lambda =2\Lambda B=\Lambda_{\mathrm{PM}}B$$

During the UV illumination, grating growth was monitored through the continuous observation of FBG transmission spectrum by the optical spectrum analyzer with 0.02 nm resolution. The optical fiber was connected with a broadband superluminescent diode through the polarization controller, which was set in such a way as to equally excite both polarization modes. Additionally, in order to maintain the polarization state, the free ends of fiber were fixed to prevent it from bending and position changing. Such polarization conditioning allowed to balance both minima in the transmission spectrum of the growing FBG. During the FBGs inscription, it was found that the arrangement of the air holes in the optical fiber in relation to the UV illuminated beam does not significantly affect the quality of the gratings; at most it slightly changes the dynamics of their growth. Thus, FBGs were written until their transmission minima exceeded −2 dB. The quality of FBGs was preserved, because the UV pattern was affected only by two air–glass boundaries in contrary to the microstructured air-hole cladding fibers, in which multiple air–glass interfaces can significantly deteriorate the interference pattern behind the phase mask and thus prevent the FBG formation [31].

### 2.4. Exemplary Spectrum

**Figure 3:**
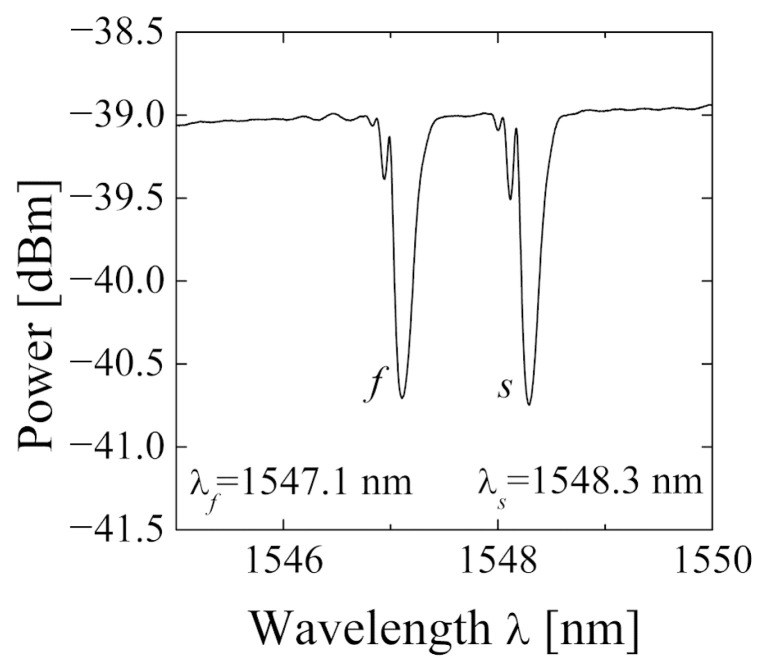
Spectral transmission characteristic of fiber Bragg gratings #1 (FBG1) written in SH2 optical fiber.

An exemplary transmission spectrum of in-written Bragg grating in SH2 optical fiber is presented in Figure 3. As mentioned above, due to the high birefringence of the optical fiber, there are two well-separated minima corresponding to the fast and slow fiber axes. At room temperature and when no strain is applied, the respective Bragg wavelengths are 1547.1 nm and 1548.3 nm, and thus their separation is 1.2 nm. Using Equation (4) the birefringence was calculated for SH2 fiber. The obtained value of 11.2 × 10^−4^ is consistent with the measured one at 1548 nm.

## 3. Sensor Characterization

**Figure 4:**
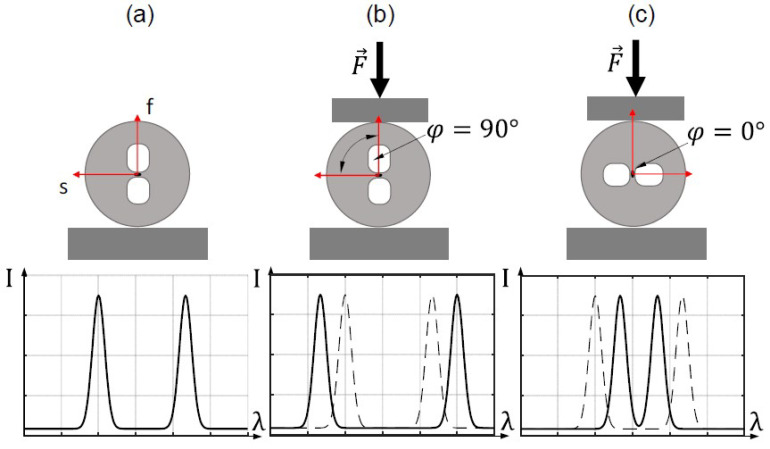
Changes in the reflection spectrum of a HiBi FBG due to an external lateral force along the main axes of the HiBi fiber (φ—angular orientation of the fiber): no load applied (**a**), load applied along (**b**) fast axis and (**c**) slow axis of the side-hole fiber.

Fiber Bragg grating inscribed in highly birefringent optical fiber shows anisotropy in load sensing abilities, especially for the radial direction (perpendicular to light propagation). This means that the Bragg wavelength sensitivity to external transversal force for both polarization modes depends on the rotation of the fiber (angular orientation) [20,21,32,33]. This effect is schematically illustrated in Figure 4. The angular orientation is given as an angle between the force direction and the slow axis of an optical fiber, so φ=0°-force applied along the slow axis, φ=90°-force applied along the fast axis.

The reflection spectrum of an unloaded FBG inscribed in HiBi fiber (Figure 4a) contains two Bragg peaks with a separation of 2ΛB. Compressive loading along the fast axis—Figure 4b (φ=90°) increases the peak separation. On the other hand, loading along the slow axis, Figure 4c (φ=0°), can cause a decrease in peak separation. However, it is worth mentioning that Figure 4 is only illustrative. The Bragg peaks behavior in particular birefringent is strictly dependent on fiber construction. In particular, it may differ for solid, side-hole and microstructured birefringent optical fibers. Moreover, within the given structure, the Bragg wavelengths shifts vs. applied force may strongly depend on the design parameters of the fiber. Consequently, due to the rotation sensitivity, the determination of the main axes of the fiber before integration into the composite is important to maintain the good measuring capabilities of the sensor.

### 3.1. Determination of the Force-Sensing Sensitivity of HiBi FBG Sensors

Calibration of Bragg gratings written in birefringent optical fibers was performed in the literature using various setups. Their overview is well presented in [34]. However, the general rule is that the optical fiber is laterally compressed in a certain angular orientation with a known force. During this process, Bragg wavelengths shifts corresponding to the fast and slow axes are recorded, and the sensitivity to the external force is determined. The procedure is repeated for angular orientations ranging φ from −90° to 90°.

**Figure 5:**
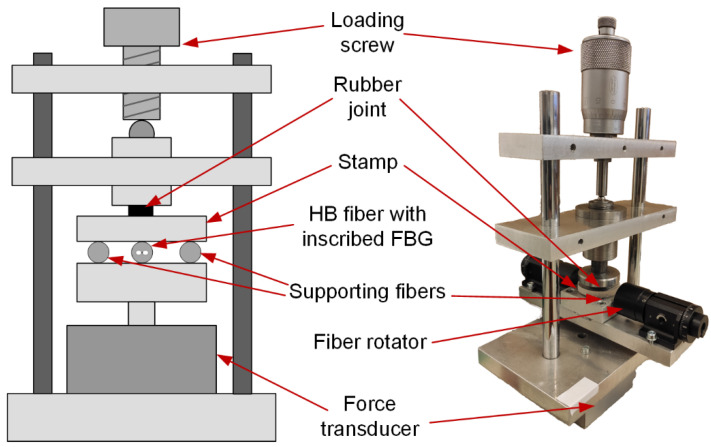
Scheme of a testing rig for the HiBi FBG testing.

For fiber Bragg gratings calibration purposes, the testing set-up was built (Figure 5). In this set-up, the loading force is applied with a screw, and then three stripped optical fibers are compressed (one with in-written FBG and two supporting). The optical fibers are positioned symmetrically in relation to the tested HiBi fiber. The lower part of the loading stamp is joined to the upper part with a circular 5 mm thick rubber roller, which makes the pressure on all-optical fibers uniform. The load is measured by a force transducer (Erichsen 1 kN) placed beneath the table with loaded optical fibers. The rotation of the examined HiBi fiber is carried out with the precise bare fiber rotators (HFR007, Thorlabs Sweden AB, Mölndal, Sweden).

Bragg wavelengths for both fast and slow peaks were derived from reflected spectrum acquired by the optical interrogator (SI405, HBM, Darmstadt, Germany). The load was assumed to be uniform along all three fibers, so the measured force was calculated into the linear load by dividing the measured force by the sum of the fiber’s lengths. Force was applied in the range of 0 to 100 N (1.67 N/mm). Characteristics of $\lambda_{\mathrm{FBG}}^{s}=\left(F,\varphi \right)$ and $\lambda_{\mathrm{FBG}}^{f}=\left(F,\varphi \right)$ for the angular orientations of the fiber from −90° to 90° with 15° steps were made. Then, linear regression was used to calculate the transverse force sensitivities of FBG for both axes ($s_{s},\;s_{f}$) for each angular orientation. Finally, the effective (differential) sensitivity ($s_{eff}$) was calculated for each angle of rotation, according to the following formula:

(6) $$s_{eff}=s_{s}-s_{f}$$

Positive $s_{eff}$ means that the peak separation increases with the increase in the transverse force for certain angular orientations of the HiBi fiber. In turn, negative $s_{eff}$ means that the Bragg peaks (corresponding to the fast and slow axes of HiBi fiber) become closer to each other as the acting force increases.

The determination of the force sensitivity does not give a straightforward answer about the transversal strain sensing ability of a particular optical fiber, but can be used as a method to determine the angular orientation, which should result in the highest strain sensing ability. In this paper, the linear force sensitivity testing of an optical fiber was performed to compare novel side-hole fiber with different optical fibers presented in the literature and to find the best orientation to integrate it into the composite. Strain sensing abilities of the optical fiber were tested after the integration into the composite samples. 

### 3.2. Transverse Force Sensitivity Curves for FBG on HiBi Fibers

In order to validate testing set-up, transverse load sensitivity vs. angular orientation of the bow-tie fiber with in-written grating was examined. Then, results can be compared with those obtained for similar bow-tie fibers with in-written gratings [20,35]. For this purpose, FBGs were written in four sections of commercially available bow-tie HiBi fiber (HB1500, Fibercore House, Southampton, UK). Then $s_{s}$, $s_{f}$, and $s_{eff}$ were derived for each angular orientation φ and characteristics of $s_{s}=f\left(\varphi \right)$, $s_{f}=f\left(\varphi \right)$ and $s_{eff}=f\left(\varphi \right)$ were plotted (basing on averaged measurement results from four in-written FBGs).

**Figure 6:**
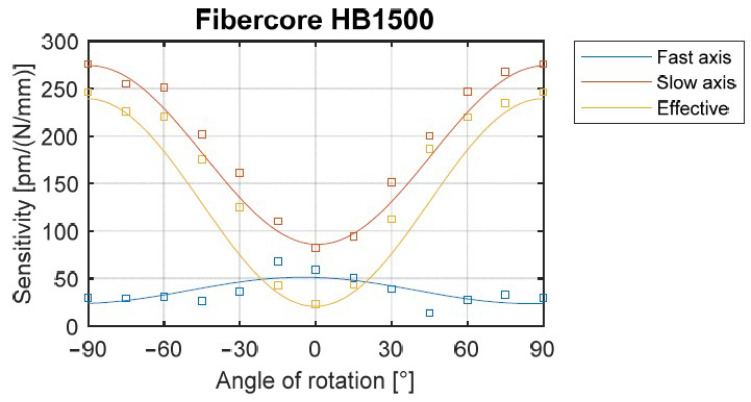
Transverse force sensitivity of the FBG sensor inscribed in the bow-tie (HB1500) fiber vs. angle of rotation.

Maximum sensitivity was observed for the angular orientation of φ=90° (when the force is applied along the fast axis). At this point, the transverse load sensitivities for f and s axes are $s_{s}=260\;\mathrm{pm}/\left(N/\mathrm{mm}\right),s_{f}=30\;\mathrm{pm}/\left(N/\mathrm{mm}\right)$, so effectively $s_{eff}=230\;\mathrm{pm}/\left(N/\mathrm{mm}\right)$. The shapes and the sensitivity values presented in Figure 6 are similar to those obtained in the previous experiments [20,35].

**Figure 7:**
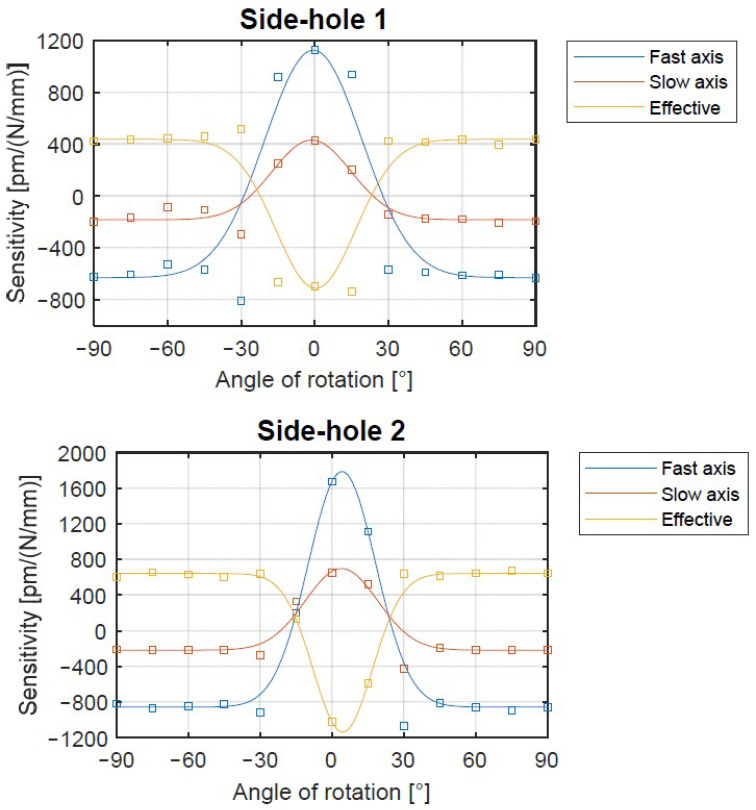
Transverse force sensitivity of the FBG sensor inscribed in side-hole fibers (SH1—**top**, SH2—**bottom**) vs. angle of rotation.

Next, transverse force sensitivity characteristics of FBG sensors written in side-hole fibers were determined in similar way. In this case the presented data are averaged from four FBGs for both SH1 and SH2 fibers (Figure 7).

Following analysis of the sensitivity characteristics of FBGs written in SH1 and SH2, it can be noticed that for both fibers, two sensitivity regions can be determined. The first one ranges from −90° to −30° and from 30° to 90°, and due to the fiber symmetry along the slow axis, can be written as ±(30–90)°. In this case $s_{s}$, $s_{f}$, and thus $s_{eff}$ are almost angular orientation φ insensitive. The second region ranging from −30° to 30° or (0 ± 30)° exhibits strong dependence of the aforementioned sensitivities with respect to the angular orientation. In this region at φ=0° the maximum effective sensitivities $s_{eff}=-700\;\mathrm{pm}/\left(N/\mathrm{mm}\right)$ and $s_{eff}=-1150\;\mathrm{pm}/\left(N/\mathrm{mm}\right)$ are noticed for both SH1 and SH2, respectively. These values (especially for SH2) are much higher than those obtained in the previous research, shown in Table 1. Negative sensitivity means that the spectral separation decreases with the increase in applied force.

On the other hand, when the SH1 or SH2 fibers are oriented at any angle within the ±(30–90)° range, the transverse force sensitivity is still competitive with the highest values obtained so far [21], and exhibit constant $s_{eff}$ value over a wide range of φ. This, in turn, ensures a high tolerance in the alignment of the optical fiber in the process of integrating the sensor with the composite structure.

Transverse load sensitivity can be negative (when the spectral separation of Bragg peaks increases) or positive (when the spectral separation of Bragg peaks decreases) versus an increase in applied force. The change in the sign of the sensitivity with the different angles of rotation is caused by the quite complex sensing mechanism. When transverse load is applied, it induces the stresses in the fiber core area in both directions (i.e., along the fast and slow axis) that are responsible for $n_{s}$ and $n_{f}$ changes due to the elasto-optic effect. The values and relation between these stresses depends on optical fiber orientation due to the existence of air holes in the fiber. For certain optical fiber positions, when the transverse load increases, the stress distribution along both fiber axes results in increasing the difference between $n_{s}$ and $n_{f}$ and thus the sensitivity is positive. Comparatively, for the other fiber arrangement, the increase in the transverse load makes the $n_{s}$ and $n_{f}$ become closer, which causes the decrease in the spectral distance between Bragg peaks and thus implies the negative sensitivity to the transverse load.

Bow-tie and side-hole fibers show different shapes in the angle-sensitivity characteristics due to the difference in their cross sections. The bow-tie fiber is made of solid material with approximately uniform stiffness (stress applying part, SAP, is made with the same glass as fiber cladding but with B dopant). Therefore, the sensitivity of birefringent solid fiber continuously changes with the rotation angle, which makes the fiber with in-written grating orientation sensitive in whole φ range. On the other hand, side-hole fibers contain large air channels located on both sides of the core. Such design causes the external transversal load to concentrate in the narrow bridge between the air holes when the force is applied along the slow axis.

To compare the sensitivities of the birefringent FBG sensors shown here, peak separation and effective sensitivity for the external linear force are collected in the Table 2.

It can be noticed that the proposed SH1 and SH2 sensors with in-written gratings exhibit very high sensitivity with relatively high spectral separation with respect to other structures. The side-hole FBG presented in [21] exhibits similar sensitivity as in the case of SH1, while SH2 sensitivity is 65% higher. What is more, very low (0.07 nm) spectral separation of Bragg peaks in side-hole FBG [21] limits its use for low transverse force values due to the spectral overlapping of both s and f spectral components. The MOF “butterfly” fiber has been reported to have very high spectral separation, even higher than SH1 and SH2. However, the transverse force sensitivity of $s_{eff}=370\;\mathrm{pm}/\left(N/\mathrm{mm}\right)$ proves that SH2 exhibits record sensitivity in relation to already known solutions.

### 3.3. Temperature Characterization of FBG Sensors Written in HiBi Fibers

Fiber Bragg gratings written in both SH1 and SH2 fibers were also characterized in terms of their temperature response within the 25–100 °C range. For both axes, similar temperature sensitivities and linear responses were obtained:

- For SH1 fiber: 10.75 pm/°C (fast axis) and 10.63 pm/°C (slow axis);
- For SH2 fiber: 10.41 pm/°C (fast axis) and 10.24 pm/°C (slow axis).

The difference between results for SH1 and SH2 fibers does not exceed 4% and is within measurement uncertainty. However, the most important fact is that temperature sensitivities corresponding to the slow and fast axes of the particular fiber are almost the same and differ by no more than 2%. This means that transverse load measurement by determining the spectral distance between Bragg wavelength peaks for the slow and fast axes is practically temperature insensitive.

## 4. Application of Fabricated FBG Sensor for Strain Measurement of Composites 

HiBi fibers with in-written FBGs have already been used for composite strain measurement [20,21,22,33]. Mostly, either a single FBG was used for transverse load measurement in composite structures [4], or two FBGs were adopted for 3-D strain sensing (e.g., [22,35]). We propose the usage of a single FBG sensor to measure the two-axial in-plane strain in a two-directional 4-point bending test.

### 4.1. Materials and Methods

**Figure 8:**
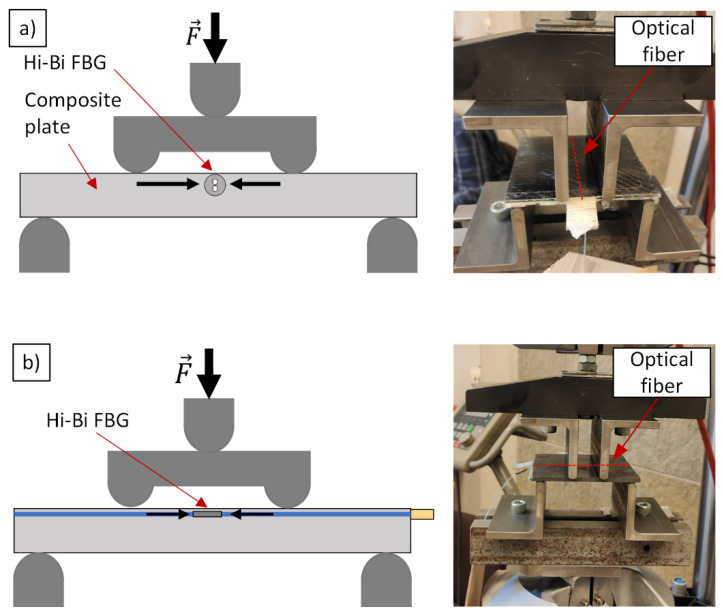
Composite samples loading scheme with embedded HiBi FBG sensors. (**a**) Compression perpendicular to the fiber; (**b**) Compression along the fiber.

Bending of a plate causes the tensile and compressive strain in some part of the sample. The further we place the sensor from the zero plane, the greater force is applied when the composite is bent. If the sensor is integrated close to the top surface, as shown in Figure 8a, it is subjected to the transversal loading, which is similar to the testing rig for sensitivity-angle determination (Figure 5). In turn, when the sample is rotated by 90 degrees, the same sensor is subjected to the axial strain (Figure 8b). Moreover, when placing the sample upside down, it is subjected to the tensile force (either axial or transversal). Considering the abovementioned configurations, the measurement capabilities of a single sensor for the multi-axial in-plane strain sensing were examined.

**Figure 9:**
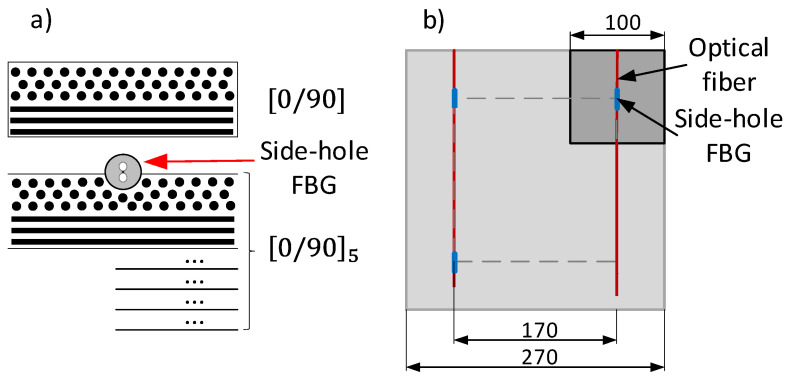
The scheme of composite samples with placed HiBi FBG sensors. (**a**) Stacking of layers and side-hole FBG placement; (**b**) Location of samples in the manufactured plate.

The proper measurement of strain with HiBi FBG is possible if the HiBi fiber is placed in the exact angular orientation. Because the diameter of a fiber is small, this orientation has to be fixed before the integration to the composite. Using the testing rig for the angle-sensitivity testing, the fiber was rotated so that the slow axis was in plane with a surface below (φ=0). This orientation was fixed with small drops of an adhesive placed on the ends of the FBG. After hardening, the flat bottom surfaces of the gluing points are in plane with the slow axis, which preserves the angular orientation during the manufacturing process. FBG sensors were integrated to the carbon fiber reinforced polymer (CFRP) in the resin transfer molding (RTM) process, as shown in Figure 9:

Plates in a size of 270 mm × 270 mm × 4 mm each, consisting of 6 layers of 0/90 carbon fiber NCF (Saertex-X-C-PB-555, with a fiber volume fraction (FVF) of 0.47 in $\;{\left[0/90\right]}_{5}/\mathrm{fib}\_90/\left[0/90\right]$ configuration were manufactured (Figure 9a). FBG sensors were fixed to the dry NCF stack with cyanoacrylate glue. The optical fiber egress points were additionally secured with PTFE tubing and silicon foam tape. The composite was saturated with Epinal 77.55-A1 resin with Epinal 77.55-B1 hardener mixed in a volume ratio of 100:32. The process was carried out under quasi-isothermal conditions at 100 °C.

After manufacturing, the plates were cut into 100 mm × 100 mm × 4 mm (Figure 9b) specimens with the FBG in the middle, near to one surface of the sample. To bend the samples, the universal testing machine MTS 810 was used. The distances between lower and higher supports were 84 and 28 mm, respectively. Strain in the depth of a sensor was calculated from the cross-head displacement.

### 4.2. Results

**Figure 10:**
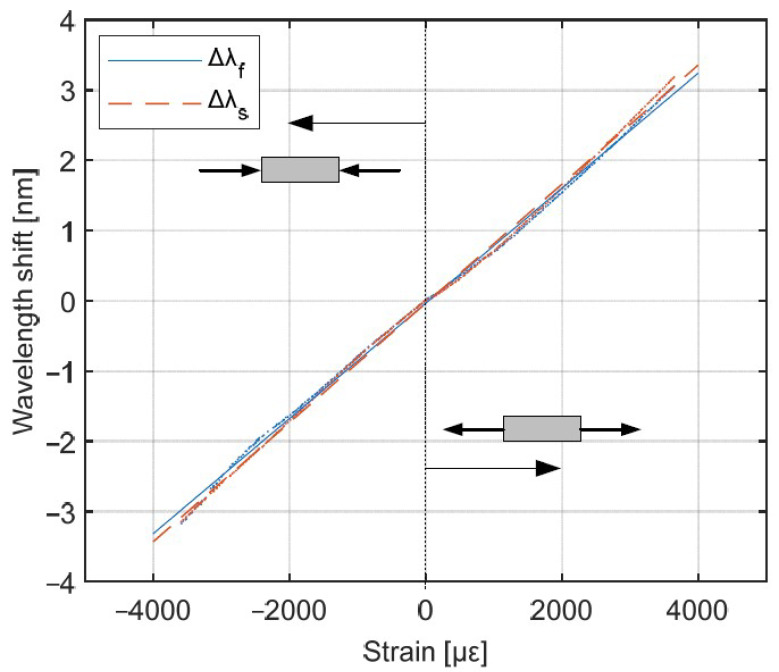
Bragg wavelengths shift of the FBG, when the strain was applied along the optical fiber.

First, the samples were examined in 4-point bending test along the optical fiber (Figure 8b). Figure 10 presents the results of the wavelength shift of both Bragg peaks with respect to the applied strain. Figures show combined results of tension and compression test along and transversal to the optical fiber.

The linear regression of the Δλ(ε) resulted in $R^{2}$ > 0.99 for both axes. The stain sensitivities derived from the linear fits of measurement data were $k_{f}=0.819\;\mathrm{pm}/\mu \varepsilon$ and $k_{s}=0.848\;\mathrm{pm}/\mu \varepsilon$ If the peak wavelength is determined with 1 pm resolution, then strain in this direction can be measured with 1.28 με resolution.

**Figure 11:**
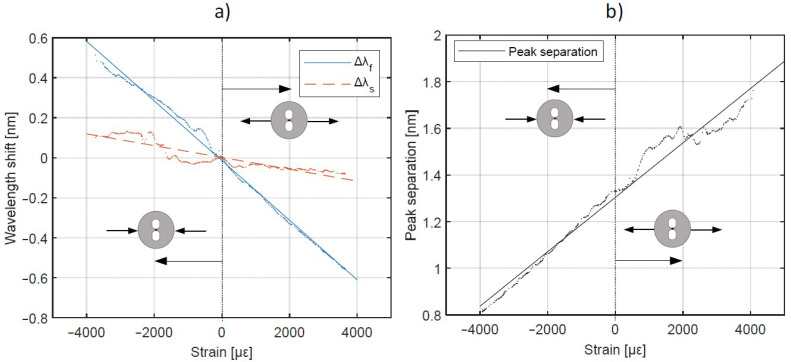
Bragg grating response for transversely applied strain: (**a**) shift and (**b**) separation of Bragg wavelengths.

Afterwards, the sample was rotated by 90° (Figure 8a), so the FBG was subjected to the transversal load. The measurement results are presented in Figure 11.

FBG subjected to the transversal strain changed its Bragg wavelengths corresponding to the fast and slow axes (Figure 11a) with sensitivities of $k_{f}=-0.149\;\mathrm{pm}/\mu \varepsilon$ $k_{s}=-0.0295\;\mathrm{pm}/\mu \varepsilon$. Peak separation changed by $k_{\mathrm{eff},⊥}=0.120\;\mathrm{pm}/\mu \varepsilon$. After linear fitting, the coefficients of determination are as follows: $R^{2}>0.99$ for the fast axis, $R^{2}=0.81$ for slow axis and $R^{2}=0.96$ for peak separation. If Bragg wavelength is measured with a resolution of 1 pm, then the resolution of the strain measurement in the transversal direction is 8.33 με.

The main reason for the inferior linear fit for Bragg wavelength changes corresponding to the slow axis (and thus worse R-squared value) is the deterioration of the Bragg peak shape after the FBG integration with the composite material. Thus, determination of the λ_B_ was more challenging. The shape of the reflected spectrum was disrupted probably by a local inhomogeneity of the composite material, which has introduced a local and directional micro-bending to the optical fiber. The other reason might be a sensor tilt in relation to the load axis. The outcome was a peak splitting observed in the reflectivity spectrum.

When the axial strain is applied, the Bragg peaks corresponding to fast and slow axis shift in the same direction and with the same sensitivity as shown in Figure 10. It means that the spectral separation does not change with the axial strain. On the other hand, when the transverse load is applied, both Bragg peaks shift, however in different ways. This is clearly shown in Figure 11, where the slopes of wavelength shift measured for slow and fast axis have significantly different values. It means that their difference (spectral separation) also changes with applied strain. Therefore, it can be stated that when using only one HiBi FBG, both transverse and axial strain can be measured. For this purpose, basing on absolute Bragg wavelength peaks and their separation derived from acquired spectrum as well as on strain sensitivities listed in Table 3, these two strain components can be calculated.

## 5. Conclusions

The number of composite structures is growing every year. Real-time evaluation of workload intensity is one of the challenges that will enable long-term and safe operation. The use of integrated fiber optic sensors allows for design and implementation of structural health monitoring systems that ensure comprehensive and continuous monitoring of composite structures from the production stage through the post-production tests to everyday use.

The paper presents both the manufacturing process of the side-hole fiber Bragg gratings and its application for measuring the multiaxial strain in composite structures. For this purpose, the specially designed highly birefringent side-hole optical fiber that had an elliptical and photosensitive core was used. Both side-hole and elliptical core ensure as high as 1.16 nm spectral separation of Bragg peaks of in-written grating, which allows for transversal force measurement even for very low values. The experimentally measured transverse load sensitivity reaches 650 pm/(N/mm) or even −1150 pm/(N/mm) depending on the fiber orientation with respect to the applied force. The proposed HiBi almost angular orientation φ insensitive within the wide range of ±(30–90)°, which makes them suitable to embedding into the composite.

Finally, the developed HiBi FBG was embedded in CFRP plates and the mechanical tests were conducted. The results show the full usefulness of the proposed solution in measuring the two-axial plane strain of composites with high sensitivity both in transverse (0.120 pm/με) and longitudinal (0.856 pm/με) directions. In the cases when plane strain measurement is sufficient, usage of these sensors can help reduce the number of optical sensors in comparison to regular FBG by half.

## Figures and Tables

**Table 1:** Geometrical parameters of SH fibers.

SH Fiber No.	Core			Glass Bridge	Side-Hole		Cladding	
	a	b	e	c	D_s_	D_f_	2R_s_	2R_f_
	[µm]	[µm]	[-]	[µm]	[µm]	[µm]	[µm]	[µm]
SH1	4.6	1.8	2.6	5.7	23.4	32.5	128.9	120.0
SH2	6.9	1.5	4.6	4.0	27.4	33.8	131.7	120.0

**Table 2:** Peak separation and maximum effective sensitivity for the transversal force for FBG sensors inscribed in different birefringent fibers.

Optical Fiber	Peak Separation [nm]	Effective Sensitivity to the Transverse Force [pm/(N/mm)]	Ref.
Panda	0.34	−210	[20]
MOF	0.85	100	[21]
MOF (Butterfly)	2.17	−370	[33]
Side-hole	0.07	699	[24]
Bow-tie-literature	0.35	160	[20,35]
Bow-tie-measured	0.32	230	This article
Side-hole 1 (SH1)	0.54	410 (φ = 90°), −700 (φ = 0°)	This article
Side-hole 2 (SH2)	1.16	650 (φ = 90°), −1150 (φ = 0°)	This article

**Table 3:** Strain sensitivities of the FBG inscribed in SH-2 fiber and integrated in the composite structure.

Load State		$k_{f}$ [pm/με]	$k_{s}$ [pm/με]	$k_{eff,⊥}$ [pm/με]
Strain along the fiber	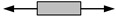 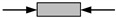	0.819	0.848	--
Strain perpendicular to the fiber	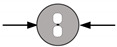 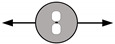	−0.149	−0.0295	0.120

## Data Availability

The data supporting this paper are available upon request by contact with the corresponding author.
